# First person – Cathryn Ugalde

**DOI:** 10.1242/dmm.043919

**Published:** 2020-01-17

## Abstract

First Person is a series of interviews with the first authors of a selection of papers published in Disease Models & Mechanisms, helping early-career researchers promote themselves alongside their papers. Cathryn Ugalde is first author on ‘Misfolded α-synuclein causes hyperactive respiration without functional deficit in live neuroblastoma cells’, published in DMM. Cathryn is a postdoctoral research scientist in the lab of Prof. Andrew Hill at La Trobe University, Bundoora, Australia, investigating neurodegeneration associated with protein misfolding.


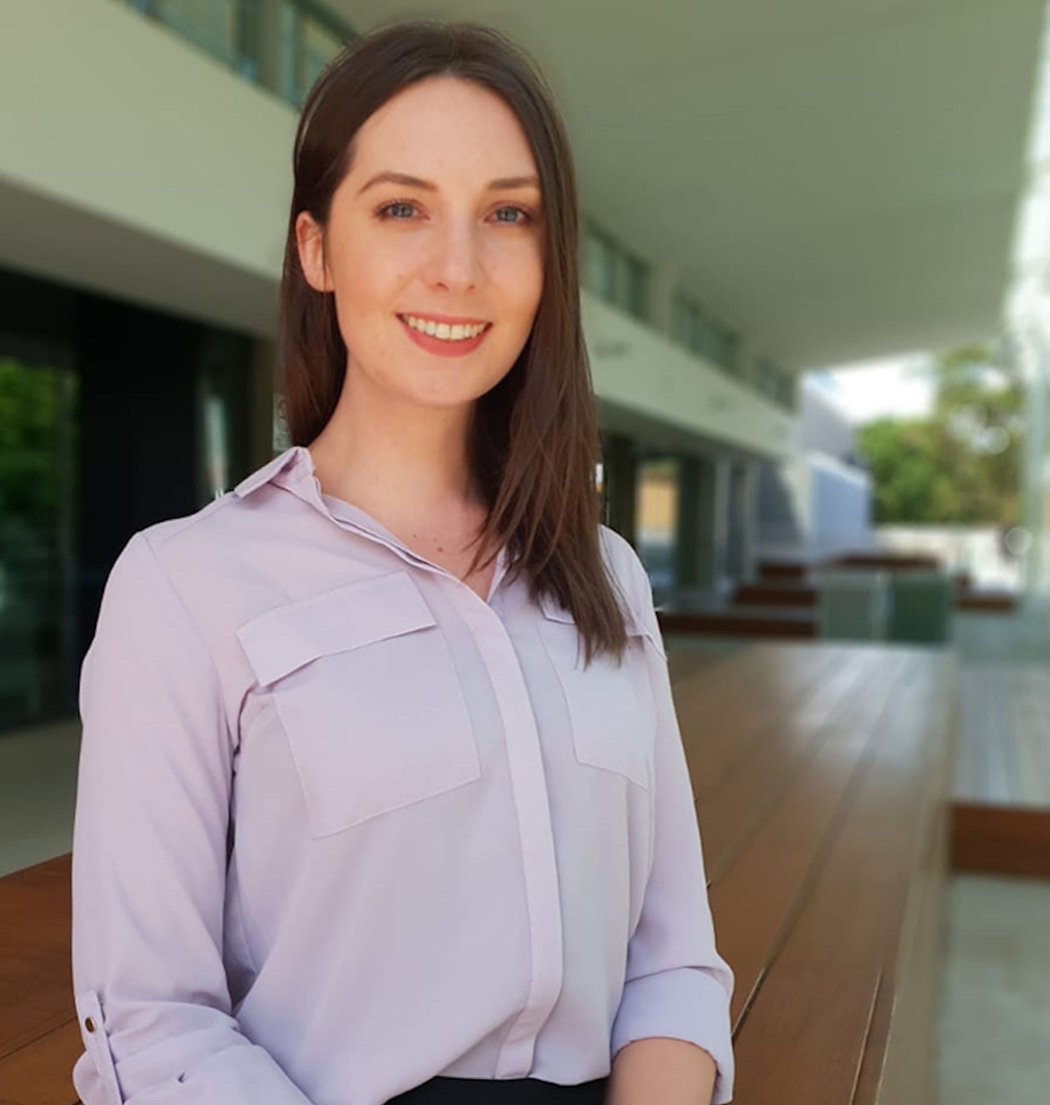


**Cathryn Ugalde**

**How would you explain the main findings of your paper to non-scientific family and friends?**

The synucleinopathies, which include Parkinson's disease, are a group of neurodegenerative disorders that are associated with misfolding of the protein α-synuclein. It is established that this misfolding event can cause the protein to adopt harmful properties that contribute to the development of disease; however, the mechanisms by which this occurs are poorly understood. This highlights the need for improved models to study the protein's pathogenicity. In this paper, we developed a new method to produce misfolded protein using an assay called protein misfolding cyclic amplification (PMCA), which involved adapting an established PMCA method to enable the production of misfolded α-synuclein to occur under non-toxic conditions. Having achieved this, we examined the pathogenic properties of the species formed by exploiting the protein's known ability to associate with lipids. Using an unbiased approach, the affinity this misfolded protein has to various classes of lipids was tested, which revealed a strong association with cardiolipin. Because cardiolipin is virtually exclusively expressed within the mitochondrion, this observation suggested that this organelle might be a target for misfolded α-synuclein. To determine if this is the case, we next measured mitochondrial respiration in live neuroblastoma cells exposed to the misfolded protein. Compared to control groups, we show that PMCA-generated misfolded α-synuclein causes hyperactive respiration without causing any specific dysfunction to the individual complexes involved in this process. This work provides evidence that hyperactive mitochondrial respiration is an important mechanism underlying the development of the synucleinopathy disorders.

**What are the potential implications of these results for your field of research?**

There are numerous studies that show that the mitochondrion is both a target for α-synuclein and is affected in models of synucleinopathy disease. However, while our work robustly shows that misfolded α-synuclein causes hyperactive respiration without functional deficit, others report that mitochondrial dysfunction occurs in models of disease. The reasons for these contrasting results are unknown but possibly stem from differences in the model used to assess mitochondrial health. In this regard, the stable transgenic systems overexpressing α-synuclein or terminal-stage disease tissues that report mitochondrial dysfunction likely harbour very different cellular environments compared to the transient system used in the current study, where misfolded protein was introduced into naïve cells. Considering our work in the context of the published literature, this may indicate that mitochondria are adaptive in disease and respond to the initial insult caused by misfolded α-synuclein via elevated respiratory outputs, before ultimately becoming overwhelmed at end-stage disease. Although much more work is needed to determine whether this may be a feature of the human condition, the cellular response caused by misfolded α-synuclein reported in our study may have important implications to our understanding of mitochondrial dynamics in the synucleinopathies.

**What are the main advantages and drawbacks of the model system you have used as it relates to the disease you are investigating?**

Within an organism, α-synuclein misfolding is highly complex and many different-sized species are present in the central nervous system in disease. Some of these are very small and highly dynamic, which has created challenges in isolating these species from organisms or producing protein with similar properties from recombinant protein in a laboratory. Indeed, traditional techniques of inducing misfolding in recombinant protein produces a homogenous population of one type of misfolded α-synuclein. The main advantage of this study is the use of PMCA, where its ability to produce a heterogeneous population of misfolded protein means that the species formed collectively may be more reflective of what is present in the human condition. The drawback to this system is an inherent limitation of using recombinant protein, whereby α-synuclein is unable to undergo any post-translational modification/s that may occur within a cell and be an important contributor to its pathogenicity *in vivo*. However, recent studies have shown the ability to produce misfolded recombinant α-synuclein harbouring post-translational modifications that are relevant in disease. These will be interesting techniques to try in future work using protein misfolded using PMCA.

**Figure:**
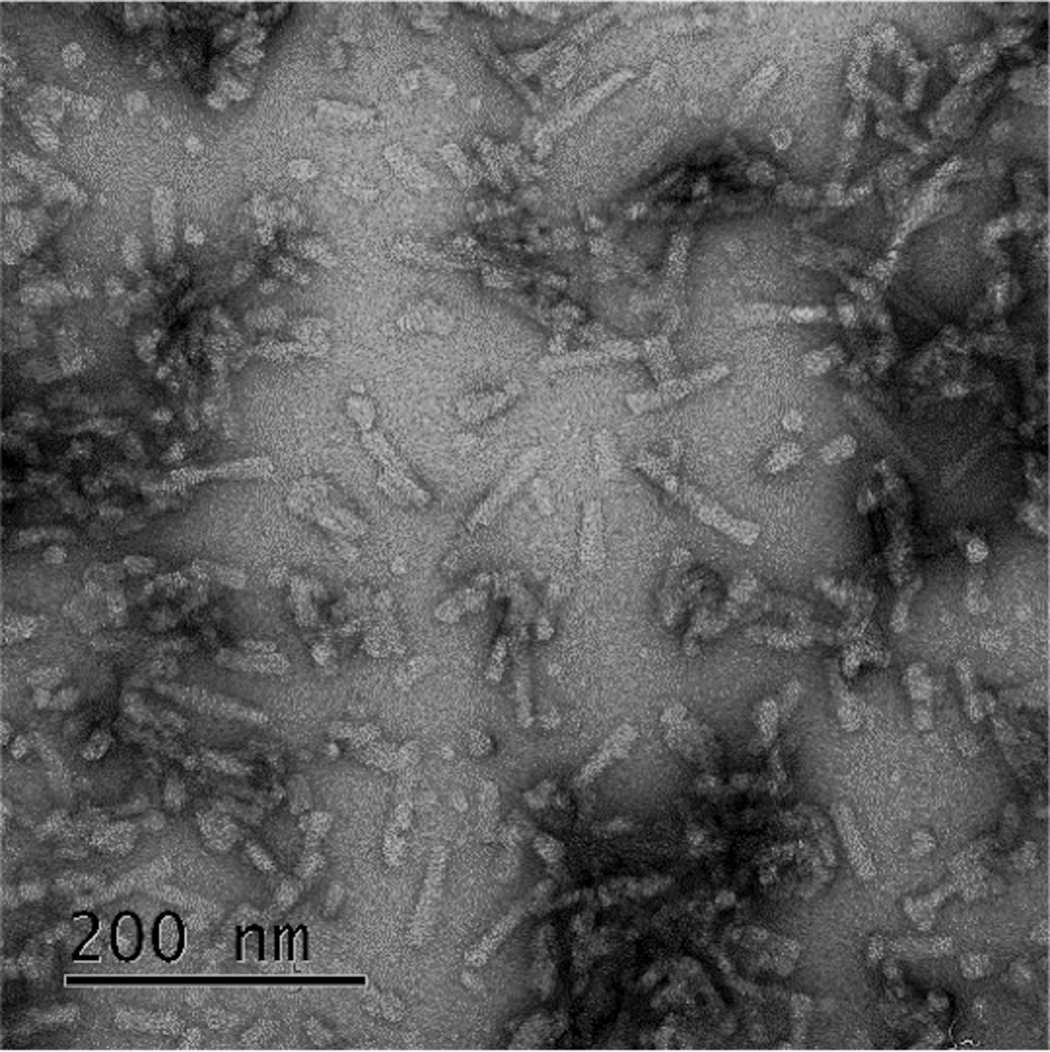
**Misfolded α-synuclein produced using protein misfolding cyclic amplification (PMCA).**

“The hyperactive mitochondrial respiration seen upon exposure to misfolded α-synuclein was a very surprising result.”

**What has surprised you the most while conducting your research?**

The hyperactive mitochondrial respiration seen upon exposure to misfolded α-synuclein was a very surprising result.

**Describe what you think is the most significant challenge impacting your research at this time and how will this be addressed over the next 10 years?**

A significant challenge in the synucleinopathy field is the complex nature of α-synuclein misfolding. Currently, it is technically challenging to accurately track and assess the generation of the various conformations of misfolded protein that may exist within an organism. These technical limitations have hindered the ability to draw strong conclusions on the most pathogenic conformation in disease. Advancements in live-cell imaging, including more sensitive protein tagging and tracking techniques, will no doubt provide important insight into these aspects of disease.

“[…] early-career researchers would benefit from more support to foster their communication skills.”

**What changes do you think could improve the professional lives of early-career scientists?**

I think early-career researchers would benefit from more support to foster their communication skills. In the current research climate, it's easy for researchers to neglect developing their soft skills to prioritize progressing their research projects. Building communication skills through things like networking, giving and receiving mentorship, and attending conferences provide so many benefits to an early-career researcher's professional skillset regardless of where their career takes them.

**What's next for you?**

Since completing my PhD, I have enjoyed being a postdoctoral scientist in the same lab in which I did my PhD, where my research interests have led me to study pathogenic mechanisms of various proteins associated with neurodegeneration. I am interested in staying in the field of neuroscience and transitioning my research towards focusing on the development of therapeutic strategies for these diseases.
